# Aggregation behavior of amphiphilic cyclodextrins in a nonpolar solvent: evidence of large-scale structures by atomistic molecular dynamics simulations and solution studies

**DOI:** 10.3762/bjoc.12.8

**Published:** 2016-01-14

**Authors:** Giuseppina Raffaini, Fabio Ganazzoli, Antonino Mazzaglia

**Affiliations:** 1 Dipartimento di Chimica, Materiali e Ingegneria Chimica ‘G. Natta’, Politecnico di Milano, via Mancinelli 7, 20131 Milano, Italy,; 2 CNR-ISMN Istituto per lo Studio dei Materiali Nanostrutturati, c/o Dipartimento di Scienze Chimiche, Biologiche, Farmaceutiche ed Ambientali dell’Università di Messina, V. le F. Stagno d'Alcontres 31, 98166 Messina, Italy

**Keywords:** aggregation, amphiphilic cyclodextrins, molecular dynamics, nanoparticles, self-assembly, simulations

## Abstract

Chemically modified cyclodextrins carrying both hydrophobic and hydrophilic substituents may form supramolecular aggregates or nanostructures of great interest. These systems have been usually investigated and characterized in water for their potential use as nanocarriers for drug delivery, but they can also aggregate in apolar solvents, as shown in the present paper through atomistic molecular dynamics simulations and dynamic light scattering measurements. The simulations, carried out with a large number of molecules in vacuo adopting an unbiased bottom-up approach, suggest the formation of bidimensional structures with characteristic length scales of the order of 10 nm, although some of these sizes are possibly affected by the assumed periodicity of the simulation cell, in particular at longer lengths. In any case, these nanostructures are stable at least from the kinetic viewpoint for relatively long times thanks to the large number of intermolecular interactions of dipolar and dispersive nature. The dynamic light scattering experiments indicate the presence of aggregates with a hydrodynamic radius of the order of 80 nm and a relatively modest polydispersity, even though smaller nanometer-sized aggregates cannot be fully ruled out. Taken together, these simulation and experimental results indicate that amphiphilically modified cyclodextrins do also form large-scale nanoaggregates even in apolar solvents.

## Introduction

**Scheme 1:**
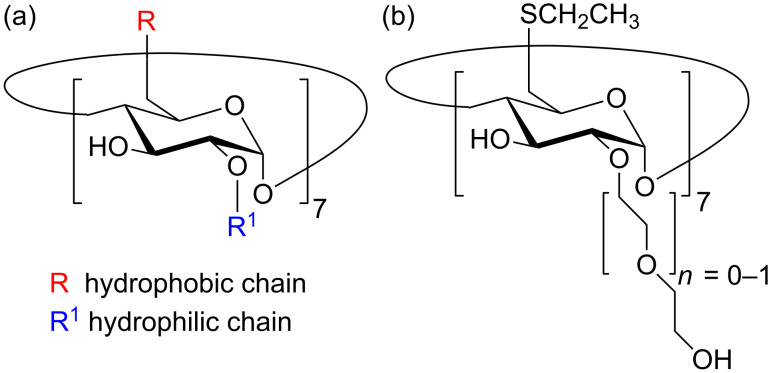
(a) Typical structure of aCD bearing hydrophobic chains (R) at the primary side and hydrophilic chain (R^1^) at secondary side; (b) peculiar structures of aCD (SC2OH) utilized in this study (*n* = 0, atomistic investigation; *n* = 1, solution studies).

Amphiphilic cyclodextrins (aCD) are a class of molecules highly investigated for their self-assembly properties and inherent potential applications [1–2]. In particular, the modification with thioalkyl chains in the primary rim and oligoethylene glycol chains in the secondary rim and, consequently, their hydrophobic–hydrophilic balance (Scheme 1) modulates the formation of differently sized and shaped nanoconstructs as membrane models for molecular recognition [3] or nanocarriers for drug delivery [4]. In the recent past, the aggregation behavior of aCD was investigated in water, getting insights on the structures of free nanoassemblies [5–6] or in combination with therapeutic agents [7]. However, in some stages of preparation and investigation of nanoconstructs based on aCD, the dissolution in organic nonpolar solvent is mandatory to prepare stable nanoemulsions [4,8], thin films [9] or organic dispersions for spectroscopic characterization of aCD unimers [10].

In a previous paper ([11], henceforth referred to as paper I) we investigated the interaction pattern of these aCD both in vacuo and in explicit water considering at first the isolated molecule, and then the interaction among two or four molecules (and some preliminary results with eight molecules) in different starting arrangement to understand the possible interaction geometries and their relative stability. In this way, we could propose various robust arrangements that could be fully stable within the simulation time at room temperature, suggesting that different metastable states could coexist together with the thermodynamically most stable ones and that kinetic control of the initial aggregation stage could easily take place. In particular, these simulations carried out with relatively small systems comprising a few molecules allowed us to assess that the driving force for aggregation was due to a synergy of different contributions. In particular, we found that the R^1^ chains (see Scheme 1: these chains were denoted as polar, or P, chains in paper I [11]) could show inclusion within the cavity of the molecule they belong to or of a neighboring molecule thanks to both dispersive and dipolar interactions and possibly with a few hydrogen bonds that may involve the glycosidic oxygen atoms. Moreover, one or two R chains (see again Scheme 1: these chains were indicated as hydrophobic, or H, chains in paper I [11]) could also show both self-inclusion and mutual inclusion within the hydrophobic cavity of their own molecule or of an adjacent one thanks to favorable dispersive interactions. We also found many cases of interactions among parts of the lateral surfaces of two or more molecules that may help forming extended structures possibly involving many more molecules than considered in paper I [11] largely due again to dispersive interactions but also to some dipolar interaction close to the secondary rim. Note that the latter interaction strength is somewhat weaker than that due to self- or mutual inclusion of a side chain in an aCD cavity, but still we found that it is strong enough to hold together a molecule to a cluster of other two or three, and that these interactions at the lateral surface could be enhanced in larger systems if the aCD is fully surrounded by other molecules. As a conclusion of this paragraph, it should also be noted however that overall we found relatively few hydrogen bonds stabilizing the aggregates of few molecules even in the simulations carried out in vacuo.

In the present paper we report full results about systems comprising eight molecules of the same aCD modelled in paper I [11] with *n* = 0 for computational simplicity, and new results about much larger systems (namely with sixty-four aCD molecules) modelled in vacuo, which can be taken as an approximation of a nonpolar and weakly interacting solvent, together with some experimental results obtained from a dichloromethane solution of SC2OH with *n* = 1 (see Scheme 1B). The pioneering knowledge acquired at this stage about the simulated aggregation of the simplest SC2OH (*n* = 0), could be utilized in perspective in a future investigation on similar aCD with *n* > 0.

In the following, we discuss our simulation and experimental results in the order, and then the simulation methodology and the dynamic light scattering procedure in the Experimental section.

## Results and Discussion

### Atomistic simulations of the formation of large aggregates

**Figure 1:**
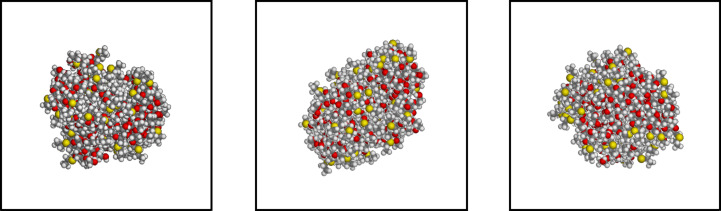
The aggregate of eight molecules of the aCD of Scheme 1 with *n* = 0, obtained from the first trial random arrangement of eight molecules in a periodic cell after the MD run of 100 ns. The three projections are taken along the cell *x*-axis, the *y*-axis, and the *z*-axis from left to right.

As summarized in the Introduction, in paper I [11] we investigated somewhat in detail the interaction pattern among two or four aCD, and mentioned some preliminary results obtained with eight molecules. Let us know describe more in detail the formation of larger aggregates that may correspond to the systems present in a nonpolar solvent, beginning with the aggregates obtained in the two independent simulations carried out with eight molecules randomly placed in vacuo in a periodic simulation cell. In either case, after the initial minimizations the eight molecules clustered in a single but still relatively loose aggregate. In the subsequent long MD run that was eventually carried out for a total of 100 ns, the two aggregates obtained by these preliminary energy minimizations turned out to be quite stable, enhancing the intermolecular interactions by relatively small local displacements, producing tighter clusters with an intermolecular interaction pattern essentially reproducing on a larger scale the pattern described in paper I [11] for two and four aCD and summarized in the Introduction. Such results were obtained for both replicas of eight molecules randomly placed in the periodic simulation cell, but the aggregate geometries turned out to be somewhat different. In fact, in one case we obtained a cluster with an elongated shape, fully isolated from its periodic images in the neighboring cells, as shown in Figure 1. In this case, the aggregate could be described as forming a prolate ellipsoid with a semi-major axis having a length of about 21.0 Å, and a semi-minor axis of about 14.5 Å. Another feature that can be noticed in Figure 1 is that the exposed hydroxy groups tend to cluster together more than the hydrophobic alkyl groups bound to the sulfur atoms (shown in yellow) because of the hydrogen bonds they form.

**Figure 2:**
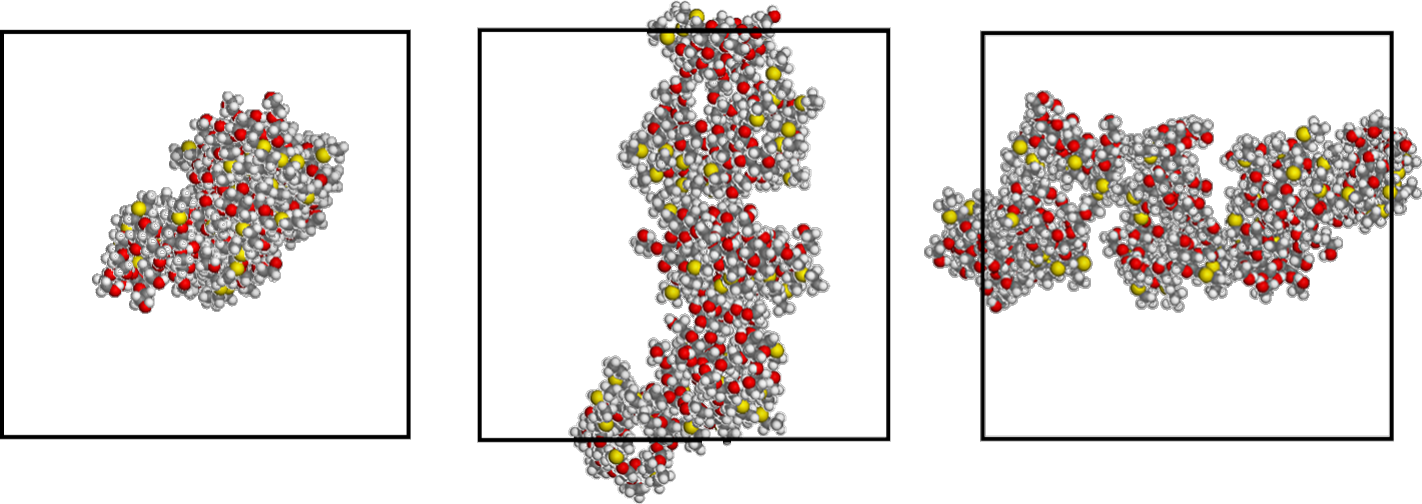
The aggregate of eight molecules of the aCD of Scheme 1 with *n* = 0, obtained from the second trial random arrangement of eight molecules in a periodic cell after the MD run of 100 ns. The three projections are taken along the cell *z*-axis, the *y*-axis, and the *x*-axis from left to right.

On the other hand, in the other case we obtained a more elongated cluster spanning the whole cell length, thus forming a continuous array along the *z* direction in the projections shown in Figure 2 if we consider its periodic images. In this case, the lateral size of the aggregate is somewhat poorly defined due to the presence of surface grooves of different size, but a good estimate of the radius of the array (if the latter is roughly viewed as a cylinder) amounts to about 14 Å. The potential energy averaged in the last part of the MD run when no change was detected in the energy components (i.e., the intramolecular terms, the van der Waals component and the electrostatic one due to the dipolar interactions) showed that the first aggregate is more stable than the other one by about 1.32 ± 0.19 MJ, where the ± sign refers to the value calculated from the standard deviations of the average potential energy values due to the thermal fluctuations. However, the very large stability of each aggregate within the MD run indicates that both are very robust conformations that may form at least for kinetic reasons and persist for a long time.

From the conformational viewpoint, it is also interesting to notice that the thioethyl chains, linked to the primary rim of the macrocycle at the C6 carbon through the sulfur atoms shown in yellow in Figure 1 and Figure 2, tend to stay at the aggregate envelope, thus allowing the hydrophilic chains to better interact among themselves through dipolar interactions and hydrogen bonds. Figure 1 and Figure 2 still show that many oxygen atoms of the polar groups, shown in red, are exposed to vacuum (or a nonpolar solvent, in practice) and thus their interactions are not completely optimized, even though the hydroxy groups comprising them may form many hydrogen bonds among themselves. On the other hand, the polar chains and the molecular hydroxy groups may become trapped at the aggregate envelope for kinetic reasons, while any large-scale rearrangement might become increasingly difficult with an increasing aggregate size.

**Figure 3:**
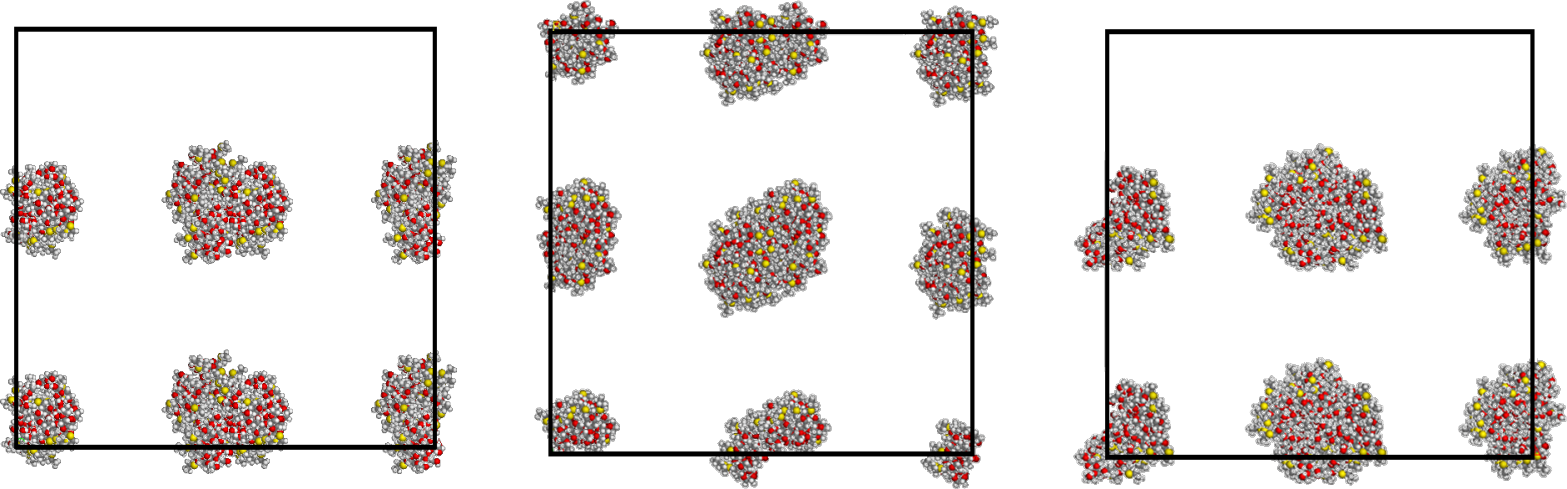
The first trial starting arrangement of sixty-four molecules within the large cell of 123.0 Å, symmetrically arranged in eight clusters each comprising eight molecules as obtained in the previous run with the smaller cell of 61.5 Å. The three projections are taken along the cell *x*-axis, the *y*-axis, and the *z*-axis from left to right. Due to the assumed cell periodicity, some clusters at the cell edges are apparently split in two smaller clusters, but they actually form eight clusters when the larger cell periodicity is taken into account.

**Figure 4:**
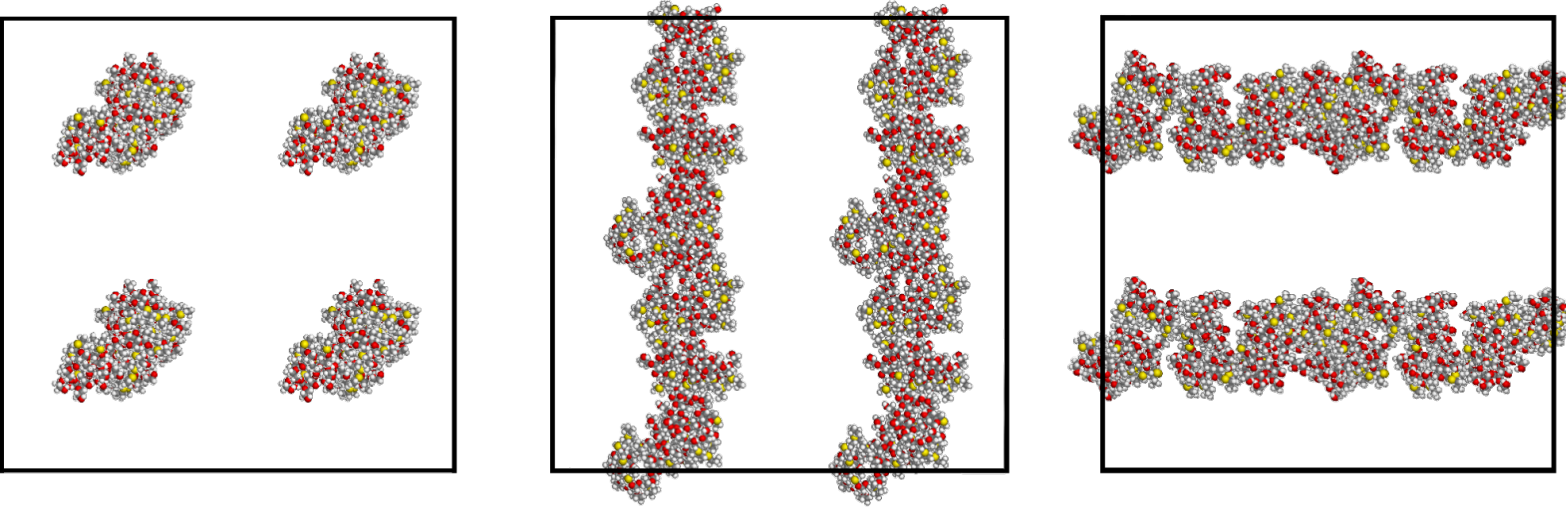
The second trial starting arrangement of sixty-four molecules within the large cell of 123.0 Å, symmetrically arranged in four parallel arrays along the *z*-axis of the projections, and each comprising twice the eight molecules of Figure 2 as obtained in the previous run with the smaller cell of 61.5 Å. The three projections are taken along the cell *z*-axis, the *y*-axis, and the *x*-axis from left to right.

In order to model larger systems, thus containing more molecules while keeping their concentration constant, we then constructed a supercell by duplicating the *x-*, *y-* and *z-*axes of the final optimized cell with eight molecules for both replicas. In this way, we obtained another cubic cell having edges of 123.0 Å and a volume equal to eight times the original one, while the total number of molecules is sixty-four, i.e., eight times their original value. These sixty-four molecules were initially related to the previous eight ones by translational symmetry, as it can be seen in the starting arrangements displayed in Figure 3 and Figure 4, but afterward they were treated as fully independent molecules.

Note that in the larger cell in one case the cluster of eight molecules is simply periodically repeated eight times, while in the other one the elongated structure initially present (see Figure 2) give rise to four parallel arrays, again repeated periodically. The preliminary energy minimization of these two systems led to only minor changes, so that the periodic repetition within the larger cell is largely preserved. Long MD simulations (50 ns) of these two systems with a total of sixty-four molecules produced significant rearrangements with the formation of extended structures with a long-range connectivity due to intermolecular non-bonded interactions again reproducing those previously mentioned.

**Figure 5:**
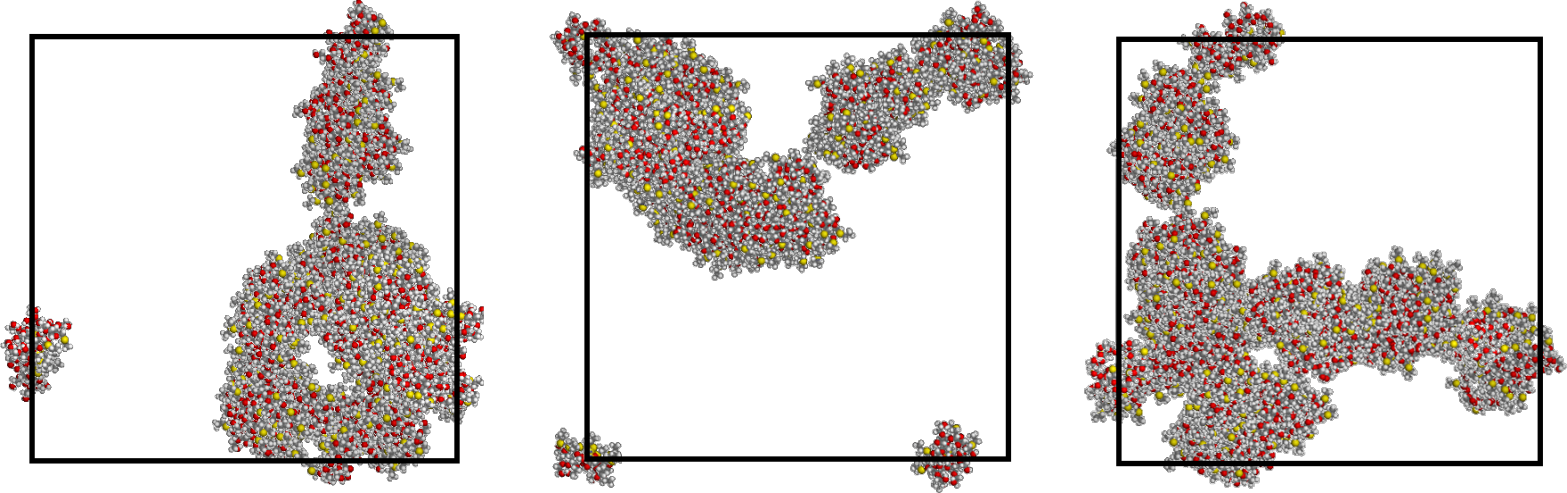
The first final arrangement of sixty-four molecules within the large periodic cell of 123.0 Å after an MD run of 50 ns viewed along the *z*-, *y*-, *x*-directions (from left to right). Due to the cell periodicity, some molecules at the cell edges are apparently separated from the whole clusters to which they belong.

The first of these extended structures is shown in Figure 5 along the three orthogonal *x*-, *y*-, *z*-directions, and considering the cell periodicity it consists of a bicontinuous structure in two dimensions with pores of different sizes that may be denoted as nanopores and mesopores. The size and possibly even the presence of these pores may be however affected by the cell periodicity, just as the ordering in arrays suggested in one case (Figure 2) for eight aCD molecules (see also Figure 4 for the starting arrangement of the sixty-four molecules that still display the previously assumed three-dimensional periodicity) yielded a more complicated bidimensional structure after the MD runs.

In this arrangement, the aggregate could be described as formed by a solid torus producing the nanopore (Figure 5 at left) and by two elongated arrays defining the mesopores, taking into account the cell periodicity. The size of this aggregate is given by the outer diameter of the solid torus of about 76 Å, and by the nanopore diameter it defines of 14 Å, whereas the apparent size of the mesopores amount to about 90 Å.

While the suggested existence of extended two-dimensional structures is supported by the simulations of the second case discussed in the next paragraph, an even larger number of independent molecules of the order of some hundreds could better clarify the issue of possible artefacts in the structural details due to the assumed periodicity. Such huge simulations are however very demanding both in terms of the number of atoms and in terms of the simulation times that would be required to let the system relax to the (pseudo) equilibrium state, so that they are in practice outside our current possibilities.

**Figure 6:**
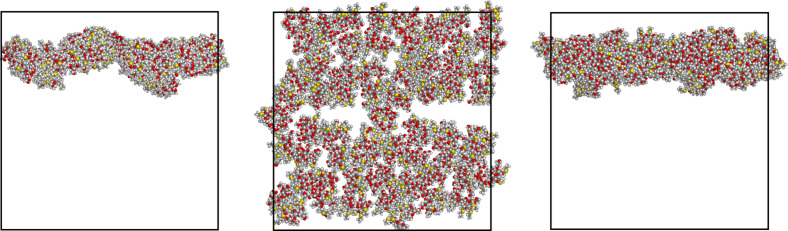
The second final arrangement of sixty-four molecules within the large periodic cell of 123.0 Å after an MD run of 50 ns viewed along the *z*-, *y*-, *x*-directions (from left to right). The flat membrane with a minor central gap is clearly seen in the central panel.

The second case mentioned before of sixty-four aCD molecules in the larger periodic cell produced at the end of the MD run the extended bidimensional structure shown in Figure 6, also in this case viewed along the three orthogonal *x-*, *y-*, *z*-directions. It is evident that in this case the initial four arrays of molecules (see Figure 4) have coalesced in two very close arrays (see the central panel of Figure 6) that may also be described as a continuous sheet in two dimensions with a minor central gap having a width of almost 10 Å, or a veritable membrane roughly parallel to one face of the periodic simulation box. The sheet thickness amounts to about 24 Å, on the average, as seen in the *x-*, *y*-projection of Figure 6 (left panel), and shows a sort of sinusoidal, or undulating profile, so that the apparent thickness appears to be larger than 34 Å in the rotated view along the *x*-axis. It should be noted that Figure 6 suggests the presence of a weakly undulating, infinite membrane. However, this pattern could also be somehow affected by the cell periodicity, and we cannot rule out the possibility that the arrangement shown in Figure 6 is but a portion of the surface of a much larger vesicle having a diameter of several tens of nanometres.

Let us finally point out that both arrangements are quite robust, in that they persist for a large part of the 50 ns MD run. On the other hand, from the thermodynamic viewpoint the calculated stability of the two arrangements of sixty-four molecules is quite different, as it can be seen for instance through the potential energy of the aggregates. Also in this case, the first aggregate shown in Figure 5 has a lower potential energy, as obtained by the values average over the last part of the MD run. The amount of overall stabilization account for 9.37 ± 0.53 MJ, where the ± sign has the same meaning as before. It is also of interest to note that the larger number of molecules induces anyway an additional stabilization due to the number of pairwise interactions that increase their number and importance more than simply proportionally to the number of molecules.

### Dynamic light scattering

**Figure 7:**
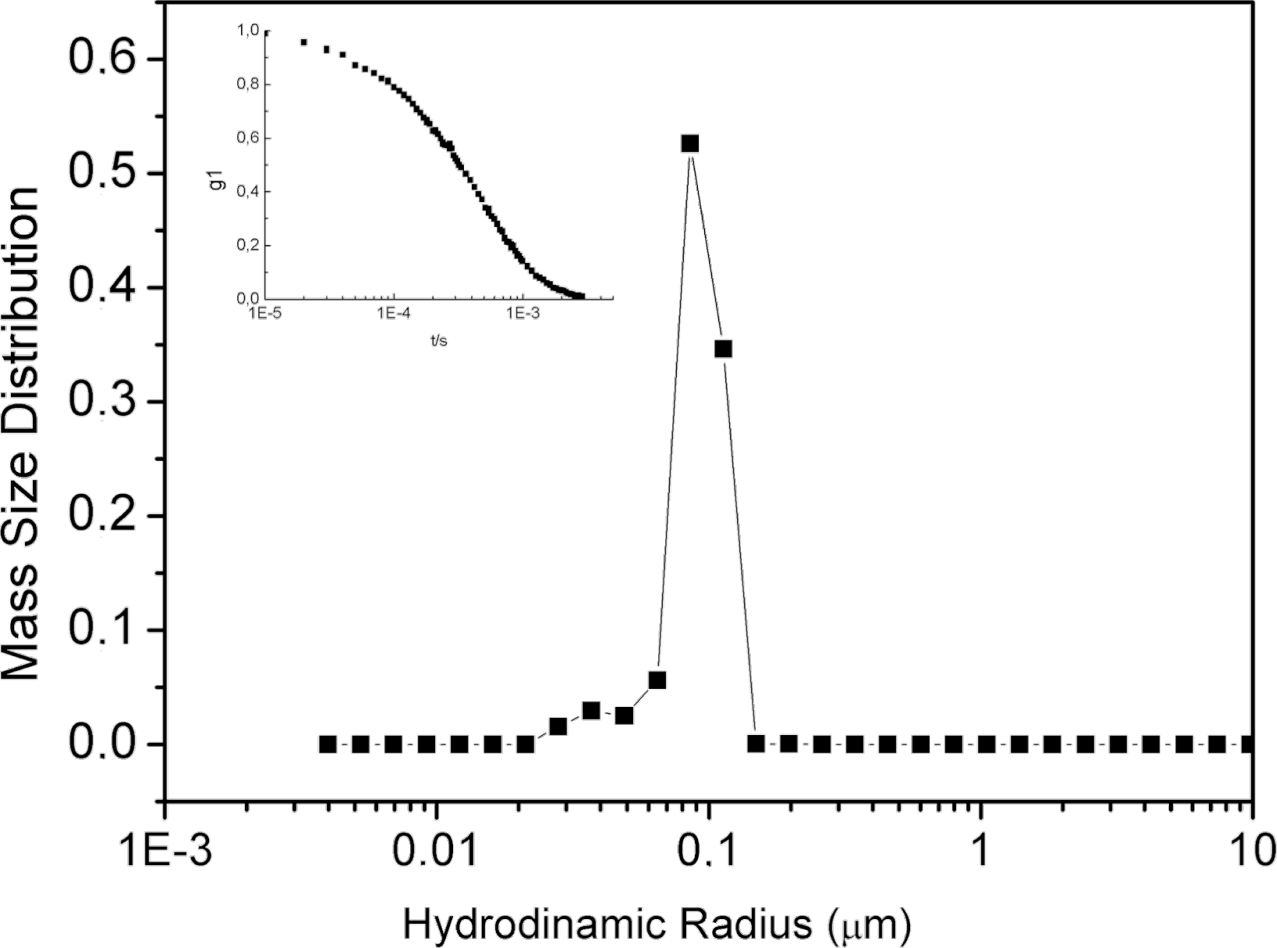
*R*_H_ distribution by CONTIN (and Mie scattering normalization) analysis of SC2OH dispersion (2 mg mL^−1^) in dichloromethane. The inset shows the correspondent scattered electric field autocorrelation function at scattering angle of 90°.

Dynamic light scattering (DLS) measurements were carried out on SC2OH (Scheme 1B, *n* = 1) dispersion in nonpolar solvent (CH_2_Cl_2_). Figure 7 shows the monomodal hydrodynamic radii distribution obtained by using CONTIN inversion algorithm and Mie scattering normalization. As a result of the cumulant analysis [12], DLS evidences the presence of aggregates with ≈80 nm hydrodynamic radius (*R*_H_) and a polydispersity index of 0.2. Furthermore, as the technique is highly sensitive to the aggregates presence, it was not possible to exclude the presence of even nanometric-sized clusters apart from the bigger aggregates [13].

## Conclusion

Amphiphilic cyclodextrins (aCD) are almost invariably investigated in a water environment for their potential use in nanomedicine, as pointed out in the Introduction, even though the use of nonpolar solvents could also be relevant for preparative or characterization purposes. In the present work, we report an investigation by means of dynamic light scattering on the aggregation properties of the compound shown in Scheme 1b (*n* = 1), dissolved in an apolar solvent and an atomistic Molecular Dynamics study of a model compound (*n* = 0 in Scheme 1) carried out in vacuo. In particular, the latter approach aims to model the bottom-up aggregation of the model aCD molecules, an approach that was started in [11], where the local details of the interaction among a few molecules were investigated. Here the atomistic modelling study takes in account an increasingly large number of molecules in vacuo, taken as an approximation of a nonpolar and weakly interacting solvent, modelled in a cell with periodic boundary conditions. Two fully independent long MD runs of increasingly large systems comprising up to sixty-four molecules suggest the presence of large-scale two-dimensional structures at length scales of more than 10 nm, comprising either relatively uniform membranes or a nano- and mesoporous mesh.

Dynamic light scattering investigations show that, indeed also in a nonpolar solvent such as dichloromethane, these amphiphilic cyclodextrins give rise to quite well defined aggregates, or nanostructures, having a hydrodynamic radius of about 80 nm and a relatively modest polydispersity. This result obtained with a slightly different compound cannot be directly compared with the results of the atomistic simulations that suggest the presence of extended bidimensional structures on scales of the order of about 10 nm, possibly affected however to some extent, or potentially even driven, by the assumed periodicity of the simulation cell having a size of 12.3 nm. However, it is interesting to speculate that the longer length of the hydrophilic chains in the experimental system may lead to strong dipolar interactions favoring the formation of larger and more compact structures compared to what predicted by the atomistic simulations. Such issue could be resolved by very long molecular simulations considering huge numbers of molecules with longer chains, unfortunately outside the current computational possibilities. As a final remark, we point out that atomistic simulations can model in detail the interaction pattern of molecules such as the amphiphilic cyclodextrins, as already noted in [11], but they can also provide a description of larger systems comprising tens or possibly a few hundreds of molecules, even though with an increasingly larger simulation burden.

## Experimental

### Simulation method

The simulations were performed with InsightII/Discover 2000 and Materials Studio [14], using the consistent valence force field CVFF [15] as previously done [16–19]. The preparation and optimization of the isolated molecule of the aCD shown in Scheme 1 (*n* = 0) was already described in paper I [11], were we also described in detail the aggregates formed by two and four molecules, and more briefly those containing eight molecules. The formation of larger aggregates discussed in the present paper was modelled by creating two independent replicas of a similar starting system obtained by randomly placing eight molecules within a cubic cell with a size of 61.5 Å using periodic boundary conditions (constant-volume conditions). After an initial geometry optimization, for both replicas the resulting adduct(s) were subjected to a long MD runs lasting for 100 ns and final geometry optimizations. In order to model even larger systems, we first duplicated the periodic cell of each replica in the three directions, obtaining a cell with twice the length of each axis (i.e., 123.0 Å), hence a volume and a number of molecules (a total of sixty-four aCD) that were both eight times as large as in the previous cell. For these larger systems, a subsequent MD run was carried out for 50 ns in each case, even though an apparent equilibration based on the time change of the potential energy was apparently achieved roughly after the first 2 ns. The dynamic equations were integrated using the Verlet algorithm with a time step of 1 fs at a temperature of 300 K, controlled through the Berendsen thermostat, and the instantaneous coordinates were periodically saved for further analysis. All the energy minimizations where carried out with the conjugate gradient method up to an energy gradient lower than 4 × 10^−3^ kJ mol^−1^ Å^−1^.

### Sample preparation and dynamic light scattering

Heptakis (2-oligo(ethylene oxide)-6-ethylthio)-β-CD (SC2OH, *M*_w_ (14EO) = 2061 Da, EO = ethylene oxide units) was synthesized in agreement with the reported procedure [20]. SC2OH was dispersed in CH_2_Cl_2_ (Romil Super Pure, Sigma-Aldrich) and studied at 2 mg mL^−1^ concentration. The dispersion was centrifuged at 3000 rpm (10 min) to remove dust and air bubbles. Dynamic light scattering (DLS) measurements were carried out on a homemade apparatus by using a duplicated Nd:YAG laser source (λ = 532.0 nm) at a power of 50 mW, at a scattering angle of 90°. The scattered light, collected in a self-beating mode, was analysed using a Malvern 4700 correlator in homodyne configuration, which builds up the normalized intensity autocorrelation function [12].
